# Reactive Oxygen Species (ROS) Drive Osteocyte Dysfunction in Diabetic Osteoporosis by Impairing Autophagy and Triggering Apoptosis

**DOI:** 10.3390/antiox14111306

**Published:** 2025-10-30

**Authors:** Mengqi Han, Minyue Zhao, Furong Bai, Mengying Wang, Bo Zhang, Jianfeng Shi, Zhongbo Liu

**Affiliations:** 1 Key Laboratory of Shaanxi Province for Craniofacial Precision Medicine Research, College of Stomatology, Xi’an Jiaotong University, Xi’an 710004, China; hanmengqi84@stu.xjtu.edu.cn (M.H.);; 2 Laboratory Center of Stomatology, College of Stomatology, Xi’an Jiaotong University, Xi’an 710004, China

**Keywords:** diabetic osteoporosis, hyperglycemia, osteocytes, oxidative stress, reactive oxygen species, autophagy, apoptosis

## Abstract

This study investigates the mechanisms underlying osteocyte injury in a high glucose (HG) environment and explores potential therapeutic targets and diagnostic markers for diabetic osteoporosis, a common complication of type 2 diabetes mellitus (T2DM). Hyperglycemia induces oxidative stress through the reactive oxygen species (ROS) production, which impair osteocytes and accelerate bone loss. To examine these effects, MLO-Y4 cells and primary mouse osteocytes were cultured under normal glucose and HG conditions, with additional treatments using N-acetylcysteine (NAC, ROS scavenger) and rapamycin (autophagy promoter and mTOR inhibitor). Cell viability, ROS levels, and the autophagy and apoptosis markers expression (Beclin1, LC3, p62, Bax, Bcl2, cytochrome C, and caspase3) were assessed using CCK8/ATP level assay, flow cytometry, Western blot, qRT-PCR, immunofluorescence, and TUNEL staining. The results showed that HG inhibits cell proliferation, induces insulin resistance, generates ROS, alters antioxidant enzymes, and promotes oxidative stress, leading to mTOR activation, subsequent autophagy inhibition, and osteocyte apoptosis. NAC mitigated these effects, while rapamycin prevented HG-induced apoptosis by inhibiting mTOR activation and promoting autophagy. This suggests that ROS-induced mTOR activation impairs autophagy and hinders the clearance of damaged osteocytes, triggering apoptosis. This research provides foundational evidence and novel insights into diabetic osteoporosis pathogenesis and potential therapies.

## 1. Introduction

Type 2 diabetes mellitus (T2DM) is a metabolic disorder characterized by insulin resistance, which is linked to an increased risk of fractures [1]. Diabetic osteoporosis is one of the most significant complications of T2DM [2,3]. Recent studies have shown that patients with T2DM exhibit more fragile trabecular and cortical bone microstructure [4,5,6,7], with the abnormalities such as imbalance of bone metabolism, retardation of bone healing, compromised osteogenic capacity, and reduction in bone quality [8,9]. However, the underlying mechanisms of bone tissue deterioration and fracture risk elevation in T2DM remain ill-defined.

Osteocytes, constituting approximately 95% of the bone cell population, are the most abundant bone cells and play a crucial role in regulating osteoblast and osteoclast activities to maintain bone quality and integrity [10,11]. Additionally, osteocytes respond to mechanical and biochemical signals [12,13]. As postmitotic cells, osteocytes viability [14,15,16] is essential for bone homeostasis. A compromised osteocyte network, with reduced numbers of viable interconnected cells, impairs the recognition of microdamage within the bone matrix [17]. Previous studies have demonstrated that T2DM can lead to decreased mechanosensing function of osteocytes [18], abnormalities in the lacunar–canalicular system [19,20], and alterations in the expression of proteins involved in bone formation and resorption [21,22]. Osteocyte dysfunction not only inhibits the lacunar–canalicular network, thereby impeding the repair of bone microcracks [23,24,25], but also disrupts the balance of bone remodeling between osteoblasts and osteoclasts [26,27,28,29]. However, the impact of hyperglycemia on osteocyte death and the underlying mechanism remains underexplored, particularly in the context of diabetic osteoporosis.

Oxidative stress is a metabolic dysfunction characterized by elevated production of reactive oxygen species (ROS) and a compromised antioxidant defense system [30]. It plays a significant role in numerous diseases, and there is increasing awareness and evidence that T2DM is tightly associated with oxidative stress through mechanisms such as lipid peroxidation, DNA damage, and mitochondrial dysfunction [31,32,33,34]. Regarding the effects of oxidative stress on diabetes and bone metabolism, in vitro studies have shown that oxidative stress inhibits osteoblastic differentiation [25,35,36,37] and induces osteoblast apoptosis [25,38]. Hyperglycemia and hyperlipidemia, two major characteristics of T2DM, have been proven to cause osteocyte dysfunction, as well as autophagy and apoptosis of osteoblast [39,40,41,42]. Furthermore, previous studies have demonstrated that high fat can lead to decreased autophagy and increased apoptosis of osteocytes due to ROS. However, whether ROS plays a role in bone cell death specifically caused by hyperglycemia remains unclear.

Our study found that in the diabetic microenvironment, elevated glucose levels lead to the excessive generation of reactive oxygen species, which in turn impair osteocyte autophagy and promote osteocyte apoptosis. This ultimately results in widespread osteocyte death and contributes to the progression of osteoporosis. Furthermore, we clarify the role of autophagy inhibition in inducing osteocyte apoptosis.

## 2. Materials and Methods

### 2.1. Cell Culture and Viability Assay

The osteocytic MLO-Y4 cell line was purchased from the Procell (Wuhan, China). Cells were cultured in α-MEM media (Hyclone, SH30265.01B, Logan, UT, USA) supplemented with 5% fetal bovine serum (FBS, Viva Cell, C04001-500, Shanghai, China) and 1% penicillin-streptomycin (Solarbio, P1400, Beijing, China) at 37 °C in 5% CO_2_ incubator. Cells were passaged upon reaching high confluency (80–90%). Briefly, after washing with PBS, cells were detached with 0.25% trypsin-ethylenediaminetetraacetic acid (EDTA) at 37 °C for 2–3 min. The reaction was stopped with complete medium containing 10% FBS, and the cells were collected by centrifugation before being reseeded. MLO-Y4 cells were plated in 96-well plates at a density of 2 × 10^3^ cells per well and allowed to adhere for 24 h. They were then treated with different concentrations of glucose for various time points, including 24 h, 48 h, and 96 h. Cell viability was assessed using a CCK-8 kit (Boster, AR1199, Wuhan, China) following the manufacturer’s instructions. Absorbance was measured at 450 nm using a spectrophotometer (BioTek, Burlington, VT, USA).

### 2.2. Experimental Grouping

The experimental groups were defined as follows. When the cells reached approximately 50% confluency, the culture medium was replaced with the respective treatment media for 96 h. To first establish the fundamental effect of high glucose, the control group (CTR) was cultured in normal glucose medium containing 5.5 mmol/L D-glucose, while the high glucose group (HG60) was cultured in medium containing 60 mmol/L D-glucose. To subsequently evaluate the role of oxidative stress, an additional HG60 + NAC group was cultured in high glucose medium supplemented with 8 mmol/L N-acetylcysteine (NAC, Beyotime, S0077, Shanghai, China). Finally, to investigate the involvement of mTOR/autophagy signaling, a further HG60 + Rapa group was cultured in high glucose medium supplemented with 50 nmol/L rapamycin (Rapa, MCE, HY-10219, Shanghai, China).

To specifically assess autophagic flux, an additional experiment using bafilomycin A1 (Baf A1) was performed. Six hours prior to the end of the 96-h treatment period, Baf A1 (50 nmol/L) was added to the cultures to inhibit autolysosomal degradation. This created Baf A1-treated and untreated subgroups for both the CTR and HG60 groups, allowing for the measurement of LC3-II turnover.

### 2.3. Measurement of Cellular ATP Levels

Cellular ATP levels were measured with an ATP Detection Kit (Beyotime, S0026, Shanghai, China) to screen the cytoprotective efficacy of NAC at concentrations of 1, 5, and 8 mM under high glucose (60 mmol/L) conditions. MLO-Y4 cells were seeded in 12-well plates (1 × 10^4^ cells/well) and, after attachment, exposed to the respective treatments for 96 h. Cells were then lysed, and the lysates were centrifuged at 12,000× *g* for 5 min at 4 °C. The resulting supernatant was mixed with the ATP detection working solution, and luminescence was recorded using a multifunctional microplate reader (BioTek, Cytation5, Burlington, VT, USA). ATP content was normalized to the total protein concentration measured by a BCA assay (Boster, AR1189, Wuhan, China) and expressed as ATP per unit protein.

### 2.4. Isolation of Primary Mouse Osteocytes

Primary osteocytes were isolated from the bone matrix of male C57BL/6 mice (approximately 25 g in weight), as previously described [43]. Briefly, after removing the soft tissue and marrow, the epiphyses of the bones were cut off, and the marrow was flushed out using PBS and a syringe. The bones were then minced into 1–2 mm chips and subjected to seven cycles of digestion: the first three cycles used 300 active U/mL collagenase (Sigma-Aldrich Corp., St. Louis, MO, USA) and the last four cycles alternated between 5 mmol/L EDTA with 1% bovine serum albumin (BSA) and 300 active U/mL collagenase. Cells obtained from the last five centrifugation cycles were cultured for 72 h in 6-well collagen-coated plates (Collagen I, rat tail) in a total volume of 2.0 mL of culture media (phenol-red-free αMEM plus 10% FBS, 1% penicillin and streptomycin, and 250 ng/mL fungizone). Subsequent experiments were performed using cells at passage 3–5, following treatment consistent with those used for the MLO-Y4 cell line. The animal experiments were conducted in accordance with the guidelines set by the Animal Care Committee of Xi’an Jiaotong University, with approval number XJTULAC2020-414.

### 2.5. ROS Measurement

MLO-Y4 cells were cultured in 6-well plates (2 × 10^4^ cells/well). After washing with PBS, the cells were incubated with DCFH-DA (10 μmol/L, Solarbio, D6470, Beijing, China) diluted in serum-free α-MEM for 25 min at 37 °C in a 5% CO_2_ incubator. The cells were then washed twice with PBS. Subsequently, the fluorescence intensity of DCF was immediately measured using a FACS Calibur flow cytometer (Agilent, NovoCyte, Beijing, China). Each sample contained 10,000 cells, with unstained MLO-Y4 cells serving as negative controls. 

### 2.6. Western Blot Analysis

Cells were washed with PBS and lysed using ice-cold lysis buffer (50 mmol/L Tris-HCl, pH 7.4, 10% CHAPS, 2 mmol/L NaF, 10 mmol/L Na pyrophosphate, 8 mmol/L β-glycerophosphate, 1 mmol/L Na orthovanadate, supplemented with Complete Protease Inhibitor Cocktail [4693159001, Roche, Basel, Basel-Stadt, Switzerland]). To assess insulin-induced Akt phosphorylation, cells were serum-starved for 30 min and then stimulated with 10 nmol/L insulin (Solarbio, I8830, Beijing, China) for 10 min before protein collection. Total cell lysates were centrifuged at 12,000 rpm for 15 min at 4 °C, and mixed with a quarter volume of 5× loading buffer. The cell lysates were then denatured at 70 °C for 10 min. Proteins were separated by 12% SDS-PAGE and transferred electrophoretically onto a PVDF membrane. The PVDF membrane was blocked with 5% BSA for 1 h at room temperature, and then incubated overnight at 4 °C with the following primary antibodies: anti-pAkt (1:1000, Cell Signaling Technology, 9271, Danvers, MA, USA), anti-Akt (1:1000, Cell Signaling Technology, 9272, Danvers, MA, USA), anti-Beclin (1:4000, Sorlarbio, K101553P, Beijing, China), anti-p62 (1:1000, Cell Signaling Technology, 5114, Danvers, MA, USA), anti-LC3 (1:1000, Proteintech, 14600-1-AP, Wuhan, China), anti-Bax (1:6000, Abways, CY5059, Shanghai, China), anti-Bcl2 (1:4000, Abways, CY5050, Shanghai, China), anti-Cytochrome c (Cyto C) (1:4000, Proteintech, 10993-1-AP, Wuhan, China), anti-mTOR (1:5000, Proteintech, 28273-1-AP, Wuhan, China), anti-p-mTOR (1:1000, Proteintech, 67778-1-Ig, Wuhan, China), anti-AMPK (1:5000, Proteintech, 66536-1-Ig, Wuhan, China), anti-p-AMPK (1:5000, Proteintech, 80209-6-RR, Wuhan, China), anti-α-Tubulin (1:40,000, Proteintech, 66031-1-Ig, Wuhan, China), anti-β-Actin (1:40,000, Proteintech, 66009-1-Ig, Wuhan, China). After washing the membrane three times with TBST, corresponding HRP-conjugated secondary antibodies (Boster, Wuhan, China, goat anti-rabbit-IgG, BA1061, goat anti-mouse-IgG, BA1062) were incubated for 2 h at room temperature. Finally, the results were visualized using an ECL kit (Biosharp, BL520B, Anhui, China) and quantified using ImageJ software, version 1.53t (National Institutes of Health, Bethesda, MD, USA). Each protein was assayed three times using different samples.

### 2.7. Quantitative Reverse Transcription PCR (qRT-PCR)

Total RNA was extracted from cells using TRIzol (SparkJade, AC0101, Dongying, China) and reverse transcribed with the PrimeScript RT Reagent Kit (SparkJade, AG0304, Dongying, China). Quantitative PCR was performed using 2×SYBR Green qPCR Mix (SparkJade, AH0104, Dongying, China), following the manufacturer’s protocol. The primer sequences used in the study are listed in Table 1. Relative mRNA expression levels were determined using the 2^−ΔΔCt^ method and normalized to Tubulin as an internal control.

### 2.8. Immunofluorescence

MLO-Y4 cells were grown on glass coverslips in 24-well plates (4 × 10^3^ cells/well) until they reached approximately 80% confluence. The cells were then fixed with 4% paraformaldehyde (Biosharp, BL539A, Hefei, China) for 30 min, and permeabilized with 0.1% Triton X-100 (Beyotime, P0096, Shanghai, China) for 5 min. After blocking with 5% BSA for 30 min, the cells were incubated overnight at 4 °C with the following primary antibodies: anti-Caspase 3 (1:100, Proteintech, 25128-1-AP, Wuhan, China), anti-LC3 (1:250, Proteintech, 14600-1-AP, Wuhan, China). The corresponding biotinylated secondary antibody (Proteintech, SA00013-2, Wuhan, China) was used for incubation. Following DAPI staining (Boster, AR1177, Wuhan, China), the coverslips were mounted on slides with anti-fade mounting medium (Boster, AR0036, Wuhan, China). Images were collected using a confocal microscope (LSM 900, Carl Zeiss, Jena, Germany).

### 2.9. TUNEL Staining

TUNEL staining (Beyotime, C1086, Shanghai, China) was performed to assess nuclear DNA fragmentation during apoptosis. For the analysis, 4 × 10^3^ MLO-Y4 cells were seeded into 24-well plates and cultured for 96 h prior to various treatments. The cells were then washed with PBS, fixed with 4% paraformaldehyde, and permeabilized with 0.3% Triton X-100. Subsequently, the cells were incubated with 100 μL of TUNEL working solution at room temperature for 60 min. DAPI was then added to stain the nuclei. TUNEL staining was performed on three independent biological replicates. For each replicate, 10 non-overlapping fields of view per sample were captured systematically using a confocal microscope (LSM 900, Carl Zeiss, Jena, Germany).

### 2.10. Statistical Analyses

All data presented are from at least three independent biological replicates (n = 3), meaning experiments were conducted on separate occasions with freshly prepared reagents and different cell passages. Data are presented as mean ± standard error of the mean (SEM). Statistical significance between groups was analyzed by one-way analysis of variance or Student’s t test using Prism 9 software (GraphPad, San Diego, CA, USA). Triplicate samples were examined per group, and experiments were repeated with similar results. Data were expressed as mean ± SEM. Results of *p* < 0.05 were considered statistically significant.

## 3. Results

- High Glucose Induces Insulin Resistance and Oxidative Stress in Osteocytes

**Figure 1:**
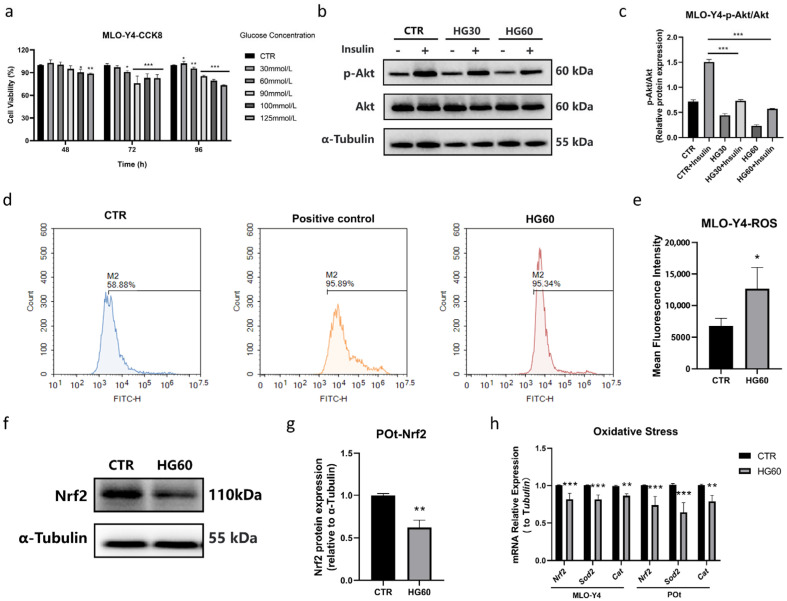
High glucose induces insulin resistance and oxidative stress in osteocytes. (**a**) Cell viability of MLO-Y4 cells treated with various concentrations of high glucose (0, 30, 60, 90, 100, 125 mmol/L) for 48, 72, and 96 h, measured using the CCK-8 assay, (**b**,**c**) representative Western blot bands and protein quantification of p-Akt and Akt in MLO-Y4 cells, (**d**,**e**) measurement of ROS levels in MLO-Y4 cells using the DCFH-DA fluorescent probe and flow cytometry, (**f**,**g**) representative Western blot bands and protein quantification of Nrf2 in primary osteocytes, (**h**) qRT-PCR analysis showing the relative mRNA expression levels of *Nrf2*, *Sod2*, and *Cat* under 60 mmol/L glucose in MLO-Y4 cells and primary osteocytes. CTR: Normal glucose. HG30: 30 mmol/L glucose. HG60: 60 mmol/L glucose. POt: Primary osteocytes. α-Tubulin was used as the loading control. Data are presented as mean ± SEM (biological replicates, n = 3). * *p* < 0.05, ** *p* < 0.01, *** *p* < 0.001 vs. CTR.

To simulate the hyperglycemic microenvironment of T2DM in vitro, MLO-Y4 cells were treated with various concentrations of high glucose (0, 30, 60, 90, 100, 125 mmol/L) for three different time points (48, 72, and 96 h). The results of the CCK8 assay demonstrated that MLO-Y4 cell proliferation was significantly inhibited by high glucose in a time- and dose-dependent manner (Figure 1a). Among the tested concentrations, 60 mmol/L high glucose notably reduced cell viability by approximately 30%. Furthermore, we investigated whether high glucose induces insulin resistance, a hallmark of T2DM. Compared to the CTR group, the expression of p-Akt/Akt was significantly decreased in the cells treated with high glucose, particularly at 60 mmol/L glucose. These results indicate dysfunction in insulin signaling (Figure 1b,c). Therefore, we chose cells treated with 60 mmol/L high glucose for further assays.

Flow cytometry analysis revealed that the ROS levels were significantly elevated after treatment with 60 mmol/L glucose compared to the CTR group, indicating increased cellular oxidative stress (Figure 1d,e). Concurrently, the protein expression level of Nrf2, a key regulator of antioxidant defense, was significantly inhibited in MLO-Y4 cells (Figure 1f,g). Additionally, the mRNA expression levels of antioxidant activity-related markers, including *Nrf2*, *Sod2*, and *Cat*, were significantly reduced in both MLO-Y4 cells and primary osteocytes (Figure 1h). These results suggest that high glucose triggers ROS generation and alters the expression of antioxidant enzymes, thereby promoting oxidative stress.

- High Glucose Activated mTOR and Inhibited Autophagy in Osteocytes

**Figure 2:**
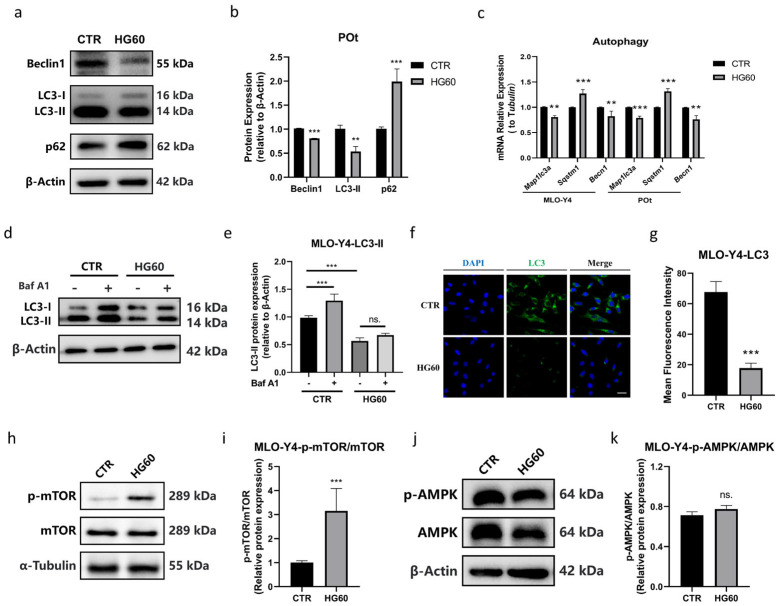
High glucose inhibited autophagy in osteocytes. (**a**,**b**) Representative Western blot bands and protein quantification of Beclin1, LC3, and p62 in primary osteocytes, (**c**) qRT-PCR analysis of the relative mRNA expression levels of *Map1lc3a*, *Sqstm1*, and *Becn1* in MLO-Y4 cells and primary osteocytes, (**d**,**e**) representative Western blot bands and protein quantification of LC3 in MLO-Y4 cells with or without Baf A1, (**f**,**g**) immunofluorescence staining showing the fluorescence intensity of LC3 in MLO-Y4 cells using a confocal microscope, (**h**,**i**) representative Western blot bands and protein quantification of p-mTOR, mTOR in MLO-Y4 cells, (**j**,**k**) representative Western blot bands and protein quantification of p-AMPK, AMPK in MLO-Y4 cells. Scale bar = 20 μm. CTR: Normal glucose. HG60: 60 mmol/L glucose. POt: Primary osteocytes. Baf A1: Bafilomycin A1. β-Actin or α-Tubulin was used as the loading control. Data are presented as mean ± SEM (biological replicates, n = 3). ** *p* < 0.01, *** *p* < 0.001 vs. CTR.

Autophagy is a highly sensitive process in response to diverse stress conditions, including high glucose and oxidative stress [44]. We first investigated whether high-glucose-induced osteocyte death is associated with autophagy dysfunction. Western blot analysis was performed to examine the protein expression of Beclin1, p62, and LC3 in both MLO-Y4 and primary osteocytes (Figure 2a,b). Compared to the CTR group, Beclin1 and LC3-II levels were significantly decreased in HG60 group, while p62 levels were significantly elevated. The mRNA expression of *Becn1*, *Sqstm1*, and *Map1lc3a* showed similar trends to the protein expression (Figure 2c). To further confirm the suppression of autophagic flux, we employed Baf A1, an inhibitor of autolysosomal degradation. Western blot analysis (Figure 2d,e) revealed that the Baf A1-induced accumulation of LC3-II was significantly attenuated in the HG60 group compared to the CTR group, which is consistent with impaired autophagic flux under high glucose conditions. In addition, immunofluorescence staining of LC3 in MLO-Y4 cells corroborated these findings, showing a significant decrease in fluorescence intensity in the HG60 group (Figure 2f,g). Given the well-established regulatory link between mTOR signaling and autophagy induction, we examined the activation of the mTOR pathway. Western blot analysis (Figure 2h,i) demonstrated that high glucose significantly increased the phosphorylation of mTOR, suggesting that autophagy suppression may be associated with mTOR hyperactivation. In contrast to mTOR, the phosphorylation levels of AMPK were not significantly altered by high glucose treatment (Figure 2j,k), suggesting that mTOR activation occurs through an AMPK-independent mechanism. These results indicated that high glucose impairs the effective clearance of damaged osteocytes by activating mTOR and consequently inhibiting autophagy.

- High glucose induced the apoptosis of osteocytes

**Figure 3:**
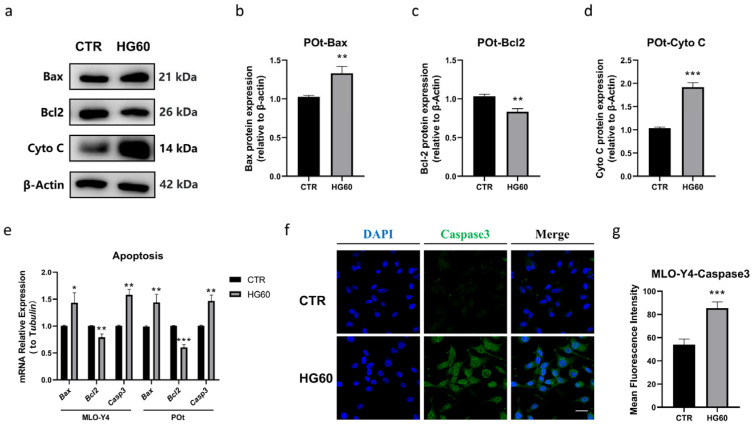
High glucose induced apoptosis in osteocytes. (**a**–**d**) Representative Western blot bands and protein quantification of Bax, Bcl2, and Cyto C in primary osteocytes, (**e**) qRT-PCR analysis showing the relative mRNA expression levels of *Bax*, *Bcl2*, *Casp 3* in MLO-Y4 cells and primary osteocytes in both groups, (**f**,**g**) immunofluorescence staining showing the fluorescence intensity of Caspase3 using a confocal microscope. Scale bar = 20 μm. CTR: Normal glucose. HG60: 60 mmol/L glucose. POt: Primary osteocytes. Cyto C: Cytochrome C. β-Actin was used as the loading control. Data are presented as mean ± SEM (biological replicates, n = 3). * *p* < 0.05, ** *p* < 0.01, *** *p* < 0.001 vs. CTR.

Apoptosis, a crucial type of programmed cell death, is closely related to high glucose levels and oxidative stress. To determine whether the high glucose treatment induces apoptosis in osteocytes, we evaluated the expression levels of apoptosis-related markers. Western blot analysis revealed that, compared with the CTR group, the expression levels of pro-apoptotic protein Bax were significantly increased, while the expression of the anti-apoptotic protein Bcl2 was decreased in HG group (Figure 3a–d). A significant upregulation was also observed in the protein expression of Cyto C, which participates in the initiation of apoptosis. Moreover, the mRNA expression levels of *Bax* and *Casp 3* (apoptosis executor) were significantly increased, whereas the mRNA expression level of *Bcl2* was decreased, consistent with the changes observed in their corresponding protein levels (Figure 3e). Similarly, immunofluorescence staining for Caspase 3 also confirmed the induction of apoptosis following HG treatment (Figure 3f,g). Taken together, these data suggest that high glucose treatment promotes apoptosis in osteocytes, leading to a significant increase in osteocytes death.

- NAC Ameliorated HG-Induced Decrease in Autophagy and Increase in Apoptosis in Osteocytes

**Figure 4:**
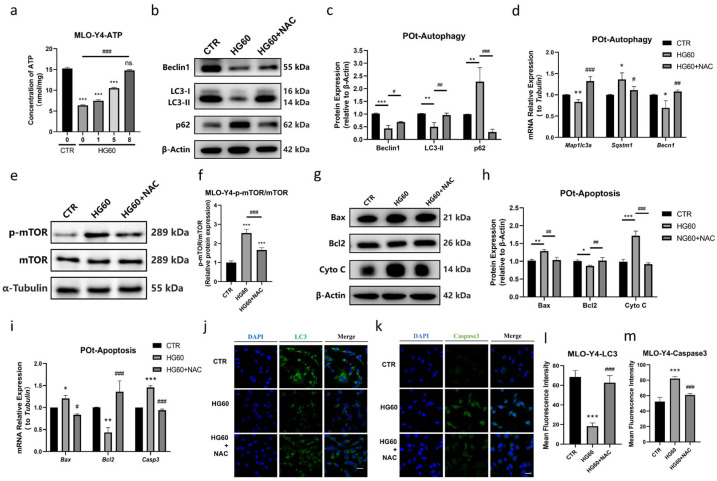
NAC ameliorated HG-induced decrease in autophagy and increase in apoptosis in osteocytes. (**a**) ATP concentration of MLO-Y4 cells treated with 60 mmol/L glucose together with NAC (0, 1, 5, 8 mmol/L) for 96 h, (**b**,**c**) representative Western blot bands and protein quantification of Beclin1, LC3, p62 in primary osteocytes, (**d**) qRT-PCR analysis showing the relative mRNA expression levels of *Map1lc3a*, *Sqstm1*, *Becn1* in primary osteocytes, (**e**,**f**) representative Western blot bands and protein quantification of p-mTOR and mTOR in MLO-Y4 cells, (**g**,**h**) representative Western blot bands and protein quantification of Bax, Bcl2, Cyto C in primary osteocytes, (**i**) qRT-PCR analysis showing the relative mRNA expression levels of *Bax*, *Bcl2*, *Casp 3* in primary osteocytes, (**j**–**m**) immunofluorescence staining showing the fluorescence intensity of LC3 and Caspase3 in MLO-Y4 cells, observed using a confocal microscope. Scale bar = 20 μm. CTR: Normal glucose. HG60: 60 mmol/L glucose. HG60 + NAC: 60 mmol/L glucose with 8 mmol/L NAC. POt: Primary osteocytes. Cyto C: Cytochrome C. β-Actin or α-Tubulin was used as the loading control. Data are presented as mean ± SEM (biological replicates, n = 3). * *p* < 0.05, ** *p* < 0.01, *** *p* < 0.001 vs. CTR. ^#^ *p* < 0.05, ^##^ *p* < 0.01, ^###^ *p* < 0.001 vs. HG60.

To investigate the potential role of ROS in the reduction in autophagy and the increase in apoptosis induced by HG in osteocytes, the ROS scavenger NAC was employed. The effects of NAC (1, 5, and 8 mmol/L) on cell viability in HG-treated MLO-Y4 cells were initially assessed using the ATP measurement. The results demonstrated a dose-dependent protective effect of NAC on ATP concentration, with a significant reversal observed at 8 mmol/L (Figure 4a). Based on these findings, 8 mmol/L NAC was selected for subsequent experiments. Firstly, the impact of ROS on the suppression of autophagy in osteocytes was evaluated. At the protein level, NAC partially reversed the high-glucose-induced inhibition of autophagy in primary osteocytes by increasing the expression of Beclin1 and LC3-II, and decreasing p62 levels, as evidenced by Western blot analysis (Figure 4b,c). Immunofluorescence staining further confirmed that the fluorescence intensity of LC3 was significantly higher in the HG60 + NAC group compared to the HG60 group (Figure 4j,l). In addition, the elevated mTOR phosphorylation induced by high glucose was significantly reversed by NAC to a level similar to the CTR group (Figure 4e,f). Furthermore, we investigated whether the AMPK pathway, a known cellular energy sensor and mTOR regulator, was involved. As shown in Figure 2j,k, high glucose did not activate AMPK. Notably, co-treatment with NAC did not rescue but slightly increased p-AMPK levels (Supplementary Figure S1), suggesting that this AMPK activation is a consequence of ROS scavenging rather than the cause of mTOR inhibition. Secondly, the role of ROS in promoting apoptosis in osteocytes was examined. Western blot analysis revealed that NAC mitigated the expression of apoptosis-related proteins by upregulating Bcl-2 and downregulating Bax and Cyto C (Figure 4g,h). This observation was strongly supported by immunofluorescence staining for caspase 3, which showed reduced apoptosis in the HG60 + NAC group (Figure 4k,m). Additionally, the effects of ROS on autophagy and apoptosis were validated at the mRNA level, with results consistent with the protein level findings (Figure 4d,i). These data collectively suggest that ROS play a crucial role in promoting mTOR activation, which subsequently leads to the dysregulation of autophagy and apoptosis in osteocytes under high glucose conditions, and that NAC effectively counteracts these effects by scavenging ROS.

- Rapamycin Prevented HG-Induced Apoptosis in Osteocytes by Promoting Autophagy

**Figure 5:**
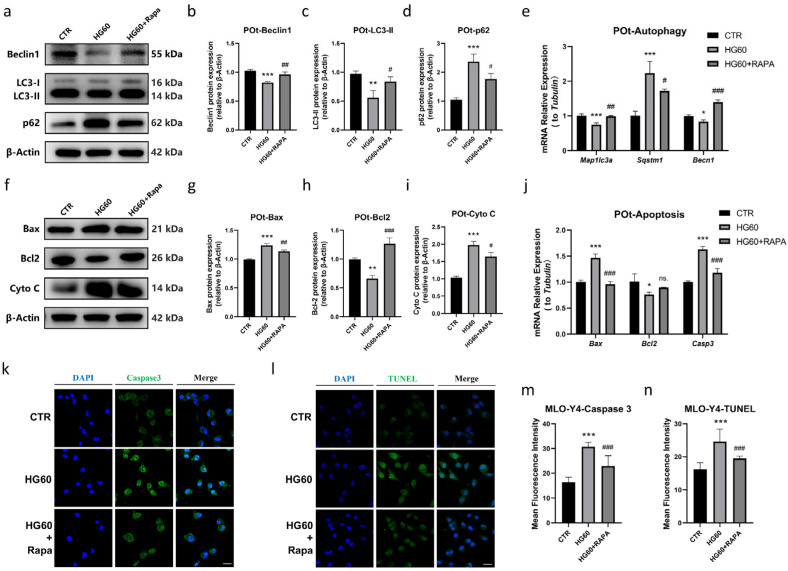
Rapamycin prevented HG-induced apoptosis in osteocytes by promoting autophagy. (**a**–**d**) Representative Western blot bands and protein quantification of Beclin1, LC3, p62 in primary osteocytes, (**e**) qRT-PCR analysis showing the relative mRNA expression levels of *Map1lc3a*, *Sqstm1*, *Becn1* in primary osteocytes in three groups, (**f**–**i**) representative Western blot bands and protein quantification of Bax, Bcl2, Cyto C in primary osteocytes, (**j**) qRT-PCR analysis showing the relative mRNA expression levels of *Bax*, *Bcl2*, *Casp 3* in primary osteocytes in three groups, (**k**–**n**) immunofluorescence staining showing the fluorescence intensity of Caspase 3 and TUNEL staining in MLO-Y4 cells, observed using a confocal microscope. CTR: Normal glucose. HG60: 60 mmol/L glucose; HG60 + Rapa: 60 mmol/L glucose with 50 nmol/L rapamycin. POt: Primary osteocytes. Cyto C: Cytochrome C. β-Actin was used as the loading control. Data are presented as mean ± SEM (biological replicates, n = 3). * *p* < 0.05, ** *p* < 0.01, *** *p* < 0.001 vs. CTR. ^#^ *p* < 0.05, ^##^ *p* < 0.01, ^###^ *p* < 0.001 vs. HG60.

As previously discussed, ROS is a key factor in inhibiting autophagy and promoting apoptosis in osteocytes under high glucose conditions. Although autophagy has been implicated as a mechanism contributing to apoptosis in certain pathological conditions, the direct relationship between autophagy and apoptosis induced by high glucose remains unclear. To investigate this connection, we employed the autophagy agonist and mTOR inhibitor rapamycin to explore the interplay between mTOR activation, autophagy and apoptosis. First, we evaluated the effects of rapamycin (50 nmol/L) on promoting autophagy by examining the protein levels of Beclin1 and LC3-II, which were significantly increased, and the protein levels of p62, which were decreased (Figure 5a–d). The mRNA expression trends were consistent with the protein data (Figure 5e). Next, we assessed whether promoting autophagy in osteocytes could mitigate apoptosis. Western blot analysis revealed that rapamycin inhibited the high-glucose-induced elevation of Bax and Cyto C protein levels, while increasing Bcl2 protein levels in the HG60 + Rapa group (Figure 5f–i). These protein level findings were supported by mRNA expression data of *Bax*, *Bcl2*, and *Casp3*, which showed significant changes compared to the HG60 group (Figure 5j). Moreover, immunofluorescence staining for Caspase 3 and TUNEL staining confirmed that rapamycin significantly decreased the expression of Caspase 3 and the fluorescence intensity of TUNEL staining, indicating decreased apoptosis in the HG60 + Rapa group (Figure 5k–n). Based on these results, we hypothesize that high glucose promotes mTOR activation, which in turn induces a decrease in autophagy in osteocytes, playing a critical role in promoting apoptosis, and that rapamycin effectively counteracts this effect by inhibiting mTOR and thereby promoting autophagy.

## 4. Discussion

**Figure 6:**
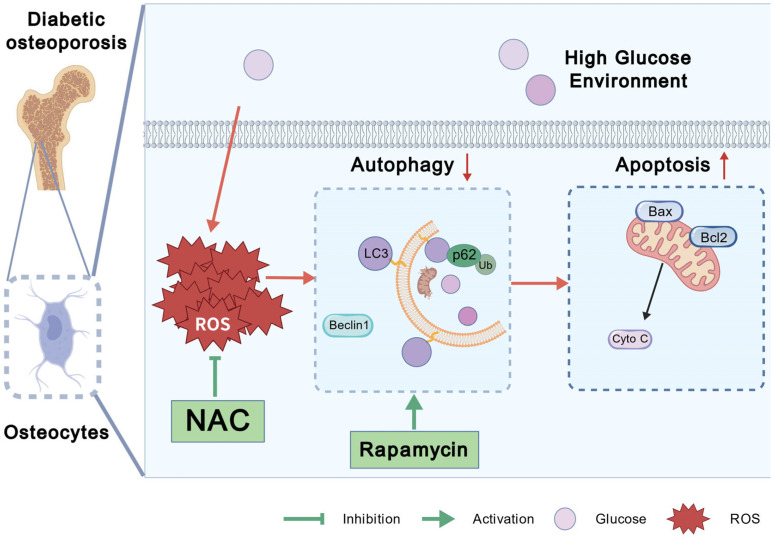
A model for high glucose-induced osteocyte dysfunction in diabetic osteoporosis through decreased autophagy and increased apoptosis. High glucose leads to excessive production of ROS in osteocytes, which activates mTOR, inhibits autophagy and subsequently induces apoptosis. The increase in osteocytes apoptosis results in osteocyte death, ultimately contributing to diabetic osteoporosis. (Created with BioGDP.com [45]).

In the current study, we observed that high glucose-induced ROS led to mTOR activation, which subsequently mediated a reduction in LC3-II, Beclin1, and Bcl2 levels, accompanied by an increase in p62, Bax, and Cyto C levels, indicating the suppression of autophagy and the enhancement of apoptosis. The administration of NAC, an ROS scavenger, reversed these effects by inhibiting mTOR activation. Additionally, rapamycin, a specific mTOR inhibitor, mitigated osteocyte apoptosis by restoring autophagy, suggesting that mTOR-mediated impairment of autophagy contributes to the promotion of apoptosis (Figure 6). Collectively, our findings offer experimental evidence supporting the exploration of potential therapeutic targets for diabetic osteoporosis.

Oxidative stress, resulting from an imbalance between oxidant and antioxidant levels, is a key factor in the onset and progression of chronic complications related to diabetes, such as nephropathy, atherosclerosis, retinopathy, and osteoporosis [46,47,48]. The accumulation of ROS is a major cause of oxidative stress and leads to a series of cellular damages [49,50]. The generation of ROS is primarily mediated by the activation of NADPH oxidase (NOX) and the enhanced activity of the mitochondrial electron transport chain, both of which are upregulated in response to hyperglycemia [51,52]. ROS disrupt the cellular redox balance and activate inflammatory responses, further amplifying cellular injury and tissue inflammation characteristic of diabetic complications [53]. In osteoblasts, excessive ROS impair osteogenic differentiation [54] and may induce ferroptosis [55] or apoptosis [56]. Consequently, targeting ROS has emerged as a promising strategy for ameliorating osteoporosis. For instance, paeonol has been shown to exert context-dependent protective effects: in age-related osteoporosis, it promotes bone formation by inhibiting NOX2-derived ROS in osteoblasts, whereas in disuse osteoporosis, it suppresses bone resorption by restoring SIRT3 expression, maintaining mitochondrial function, and enhancing SOD2-mediated ROS clearance in osteoclasts [57]. Other agents such as metformin [58], SF/HA hybrid coated titanium implant [59], and gastrodin [60], have also demonstrated osteoprotective effects via ROS scavenging. In line with these findings, our study further confirms that ROS accumulation directly impairs osteocyte viability. These results underscore the critical role of ROS in driving osteocyte dysfunction and highlight ROS clearance as a potential therapeutic target for preserving osteocyte health in diabetic osteoporosis.

In addition, the overgeneration of ROS contributes to diabetic complications by interfering with programmed cell death. T2DM patients suffer from multifactorial onset of beta-cell dysfunction and/or loss of beta-cell mass owing to ROS production, mitochondrial dysfunction and autophagy [61]. Autophagy is a critical cellular process for maintaining homeostasis [62]. ROS can trigger autophagy through the AMPK-mTOR signaling axis. Specifically, ROS activate AMPK, which in turn suppresses mTOR activity via TSC2 regulation [63]. This AMPK activation and mTOR inhibition collectively promote autophagy initiation, partly through phosphorylation of key autophagy-related proteins such as ULK1, ATG13, and AMBRA1—processes known to facilitate autophagosome nucleation. Conversely, mTOR activation has been shown to phosphorylate and inhibit components of the autophagy machinery, thereby suppressing autophagic flux [64]. Building upon this established mechanism, our findings demonstrate that high glucose-induced ROS accumulation activates the mTOR signaling pathway in osteocytes. Specifically, we observed that hyperglycemia significantly promotes mTOR phosphorylation, which subsequently suppresses autophagic activity and impairs autophagic flux. This demonstrates an mTOR-driven inhibition of autophagy. Our results thus validate the critical role of the ROS–mTOR–autophagy axis in osteocyte dysfunction under diabetic conditions and provide experimental evidence supporting mTOR as a potential therapeutic target for diabetic osteoporosis.

Additionally, ROS also plays a pivotal role in the induction of apoptosis, particularly under hyperglycemic conditions, where the mitochondrial pathway is a central mechanism [65]. ROS can directly oxidize and inactivate anti-apoptotic proteins such as Bcl-2, while promoting the activation of pro-apoptotic proteins like Bax [66]. This imbalance leads to the permeabilization of the mitochondrial outer membrane, and the release of Cyto C into the cytosol. Cyto C then activates the caspase cascade, ultimately leading to apoptosis [67]. In addition, ROS can trigger endoplasmic reticulum stress and activate death receptor pathways, further promoting apoptosis [68]. In the hyperglycemic microenvironment, autophagy and apoptosis form a tightly intertwined and complex regulatory network that profoundly influences cell fate [69,70,71]. Impaired autophagy under high glucose conditions results in the accumulation of damaged mitochondria, aggregated proteins, and other cellular debris, which can trigger apoptosis [72]. The accumulation of these toxic substances activates the mitochondrial pathway of apoptosis by increasing the production of ROS and promoting the release of Cyto C from the mitochondria [67]. Conversely, the onset of apoptosis can feed back to modulate autophagy. It has been proved that the crosstalk between autophagy and apoptosis is partially mediated by the interaction between Beclin1 and Bcl-2. In the present study, we similarly demonstrated that excessive ROS generation leads to increased osteocyte apoptosis. Furthermore, through interventions with NAC (a ROS scavenger) and rapamycin (an mTOR inhibitor and autophagy inducer), we confirmed that this pro-apoptotic effect is mediated by ROS-induced mTOR activation and subsequent suppression of autophagy in osteocytes. Therefore, under hyperglycemia, ROS activates mTOR, inhibits osteocyte autophagy, and induces osteocyte apoptosis, resulting in osteocyte dysfunction and diabetic osteoporosis.

Previous studies have proposed several biomarkers for osteoporosis diagnosis, such as bone turnover markers, N-terminal propeptide of type I procollagen (PINP), and C-telopeptide of type I collagen (CTX-I), which can be used as auxiliary indices to evaluate drug response and treatment compliance in osteoporosis patients [73]. In this study, we determined that high glucose could induce dysregulation of autophagy and apoptosis in osteocytes, suggesting potential biomarkers for the early diagnosis of diabetic osteoporosis. Additionally, exploring the dynamic changes in autophagy- and apoptosis-related molecules released by osteocytes in blood, urine, or bone microenvironments can provide new directions and methods for the early diagnosis of diabetic osteoporosis. Previous studies have used serum biomarkers as indicator for bone loss, including estrogen, calcium, vitamin D3, and alkaline phosphatase (ALP) [74]. Furthermore, based on the findings of this study demonstrating the effects of ROS on autophagy and apoptosis of osteocytes under high glucose conditions, this study may offer new drug targets and intervention strategies for the treatment of diabetic osteoporosis. In recent years, natural pharmaceutical active ingredients have gained attention as an important direction in drug discovery due to their unique advantages, including high biocompatibility, multi-target effects, low drug resistance, and abundant resources, such as ginsenoside Rg3 [75] and resveratrol [76]. Therefore, it is important to identify natural active compounds that can alleviate the damaging effects of high glucose on osteocytes and improve bone destruction in diabetic osteoporosis patients.

Several limitations of this study should be acknowledged. First, our findings are derived exclusively from in vitro experiments. Future studies utilizing diabetic mouse models are necessary to validate the effects of high glucose on osteocyte autophagy and apoptosis in vivo. Furthermore, genetic approaches employing transgenic mice with modulated ROS production, autophagy, or apoptosis pathways would help substantiate the mechanistic insights gained from our cellular studies. Second, while we established the involvement of the ROS–mTOR–autophagy axis, the precise molecular mechanisms remain incompletely elucidated. Specifically, how ROS activates mTOR, the exact downstream pathways through which mTOR suppresses autophagy, and how impaired autophagy specifically triggers apoptosis in osteocytes require further investigation. Finally, the use of a high glucose concentration (60 mmol/L) to induce a stable phenotypic response in vitro may exceed physiological levels observed in diabetic patients. Consequently, further research under conditions that more closely mimic in vivo glycemic fluctuations is warranted to confirm the pathological relevance of our findings.

## 5. Conclusions

In conclusion, oxidative stress induced by high glucose inhibits osteocytes autophagy and subsequently enhances osteocyte apoptosis, ultimately contributing to the development of diabetic osteoporosis. However, this study on the underlying mechanisms remains limited in depth and scope. In the future, it will be essential to employ advanced imaging technologies, appropriate gene-edited diabetic animal models, osteocyte-specific conditional knockout animal models, and multi-omics analysis to explore the detailed molecular mechanisms of osteocyte death in diabetic osteoporosis.

## Figures and Tables

**Table 1:** List of qRT-PCR primers.

Genes	Forward Primer Sequence	Reverse Primer Sequence
*Nrf2*	CTTTAGTCAGCGACAGAAGGAC	AGGCATCTTGTTTGGGAATGTG
*Sod2*	AGGCTCTGGCCAAGGGAGAT	CACGCTTGATAGCCTCCAGCA
*Cat*	CGCTGTAGATGTGAAACGCT	TCTCCTCCTCGTTCAACACC
*Becn1*	AGGCTGAGGCGGAGAGATTG	TGTGGAAGGTGGCATTGAAGAC
*Sqstm1*	GAACACAGCAAGCTCATCTTTC	AAAGTGTCCATGTTTCAGCTTC
*Map1lc3a*	GACGGCTTCCTGTACATGGTTT	TGGAGTCTTACACAGCCATTGC
*Bax*	GAAGCTGAGCGAGTGTCTCCGGC	TCAGCTGCCACCCGGAAGAAG
*Bcl2*	CAACACTCCCTCTTGACCTATGC	GAAAATGTTCCCAAGTGAGTTAGA
*Casp 3*	CTGGAGAAATTCAAAGGACGGG	TGAGCATGGACACAATACACGG
*Tubulin*	TGACTCCTTCAACACCTTCTTCA	GATCTCCTTGCCAATGGTGTAGT

## Data Availability

The original contributions presented in this study are included in the article and Supplementary Materials. Further inquiries can be directed to the corresponding authors.

## Supplementary Materials

The following supporting information can be downloaded at: https://www.mdpi.com/article/10.3390/antiox14111306/s1, Figure S1: Representative Western blot bands and protein quantification of p-AMPK and AMPK in MLO-Y4 cells. CTR: Normal glucose. HG60: 60 mmol/L glucose. HG60 + NAC: 60 mmol/L glucose with 8 mmol/L NAC. β-Actin was used as the loading control. Data are presented as mean ± SEM (biological replicates, n = 3). *** *p* < 0.001 vs. CTR, ^###^ *p* < 0.001 vs. HG60.

## Abbreviations

The following abbreviations are used in this manuscript:

T2DM	Type 2 diabetes mellitus
ROS	Reactive oxygen species
HG	High glucose
NAC	N-acetylcysteine
EDTA	ethylenediaminetetraacetic acid
FBS	fetal bovine serum
Baf A1	bafilomycin A1
BSA	bovine serum albumin
Cyto C	Cytochrome C
POt	Primary osteocytes

## References

[1] Yamamoto M., Yamaguchi T., Yamauchi M., Kaji H., Sugimoto T. (2009). Diabetic patients have an increased risk of vertebral fractures independent of BMD or diabetic complications. J. Bone Miner. Res..

[2] Huang X., Li S., Lu W., Xiong L. (2022). Metformin activates Wnt/beta-catenin for the treatment of diabetic osteoporosis. BMC Endocr. Disord..

[3] Jiang F., Shan H., Pan C., Zhou Z., Cui K., Chen Y., Zhong H., Lin Z., Wang N., Yan L. (2019). ATP6V1H facilitates osteogenic differentiation in MC3T3-E1 cells via Akt/GSK3beta signaling pathway. Organogenesis.

[4] Shu A., Yin M.T., Stein E., Cremers S., Dworakowski E., Ives R., Rubin M.R. (2012). Bone structure and turnover in type 2 diabetes mellitus. Osteoporos. Int..

[5] Nilsson A.G., Sundh D., Johansson L., Nilsson M., Mellstrom D., Rudang R., Zoulakis M., Wallander M., Darelid A., Lorentzon M. (2017). Type 2 Diabetes Mellitus Is Associated with Better Bone Microarchitecture But Lower Bone Material Strength and Poorer Physical Function in Elderly Women: A Population-Based Study. J. Bone Miner. Res..

[6] Zeitoun D., Caliaperoumal G., Bensidhoum M., Constans J.M., Anagnostou F., Bousson V. (2019). Microcomputed tomography of the femur of diabetic rats: Alterations of trabecular and cortical bone microarchitecture and vasculature-a feasibility study. Eur. Radiol. Exp..

[7] Giner M., Miranda C., Vazquez-Gamez M.A., Altea-Manzano P., Miranda M.J., Casado-Diaz A., Perez-Cano R., Montoya-Garcia M.J. (2021). Microstructural and Strength Changes in Trabecular Bone in Elderly Patients with Type 2 Diabetes Mellitus. Diagnostics.

[8] Jiao H., Xiao E., Graves D.T. (2015). Diabetes and Its Effect on Bone and Fracture Healing. Curr. Osteoporos. Rep..

[9] Farr J.N., Khosla S. (2016). Determinants of bone strength and quality in diabetes mellitus in humans. Bone.

[10] Delgado-Calle J., Bellido T. (2022). The osteocyte as a signaling cell. Physiol. Rev..

[11] Moharrer Y., Boerckel J.D. (2021). Tunnels in the rock: Dynamics of osteocyte morphogenesis. Bone.

[12] Dallas S.L., Prideaux M., Bonewald L.F. (2013). The osteocyte: An endocrine cell … and more. Endocr. Rev..

[13] Plotkin L.I., Bellido T. (2016). Osteocytic signalling pathways as therapeutic targets for bone fragility. Nat. Rev. Endocrinol..

[14] Busse B., Djonic D., Milovanovic P., Hahn M., Puschel K., Ritchie R.O., Djuric M., Amling M. (2010). Decrease in the osteocyte lacunar density accompanied by hypermineralized lacunar occlusion reveals failure and delay of remodeling in aged human bone. Aging Cell.

[15] Choi J.U.A., Kijas A.W., Lauko J., Rowan A.E. (2021). The Mechanosensory Role of Osteocytes and Implications for Bone Health and Disease States. Front. Cell. Dev. Biol..

[16] Yvanoff C., Willaert R.G. (2022). Development of bone cell microarrays in microfluidic chips for studying osteocyte-osteoblast communication under fluid flow mechanical loading. Biofabrication.

[17] Qiu S., Rao D.S., Fyhrie D.P., Palnitkar S., Parfitt A.M. (2005). The morphological association between microcracks and osteocyte lacunae in human cortical bone. Bone.

[18] Shao X., Tian Y., Liu J., Yan Z., Ding Y., Hao X., Wang D., Shen L., Luo E., Guo X.E. (2024). Rescuing SERCA2 pump deficiency improves bone mechano-responsiveness in type 2 diabetes by shaping osteocyte calcium dynamics. Nat. Commun..

[19] Bolger M.W., Tekkey T., Kohn D.H. (2024). Peripheral canalicular branching is decreased in streptozotocin-induced diabetes and correlates with decreased whole-bone ultimate load and perilacunar elastic work. JBMR Plus.

[20] Yao Q., Tsuboi K., Hongo H., Sakakibara M., Yamamoto T., Haraguchi-Kitakamae M., Ishizu H., Liu X., Shi Y., Li W. (2025). Histochemical assessment of the femora of spontaneously diabetic torii-lepr(fa) (SDT-fa/fa) rats that mimic type II diabetes. J. Oral. Biosci..

[21] Eom Y.S., Gwon A.R., Kwak K.M., Kim J.Y., Yu S.H., Lee S., Kim Y.S., Park I.B., Kim K.W., Lee K. (2016). Protective Effects of Vildagliptin against Pioglitazone-Induced Bone Loss in Type 2 Diabetic Rats. PLoS ONE.

[22] Zhang Y., Li X., Zhou R., Lin A., Cao M., Lyu Q., Luo P., Zhang J. (2021). Glycogen Metabolism Predicts the Efficacy of Immunotherapy for Urothelial Carcinoma. Front. Pharmacol..

[23] Weinstein R.S., Nicholas R.W., Manolagas S.C. (2000). Apoptosis of osteocytes in glucocorticoid-induced osteonecrosis of the hip. J. Clin. Endocrinol. Metab..

[24] Milovanovic P., Busse B. (2023). Micropetrosis: Osteocyte Lacunar Mineralization in Aging and Disease. Curr. Osteoporos. Rep..

[25] Zhong C., Zeng X., Yi X., Yang Y., Hu J., Yin R., Chen X. (2025). The Function of Myostatin in Ameliorating Bone Metabolism Abnormalities in Individuals with Type 2 Diabetes Mellitus by Exercise. Curr. Issues Mol. Biol..

[26] O’Brien C.A., Nakashima T., Takayanagi H. (2013). Osteocyte control of osteoclastogenesis. Bone.

[27] Kitaura H., Marahleh A., Ohori F., Noguchi T., Shen W.R., Qi J., Nara Y., Pramusita A., Kinjo R., Mizoguchi I. (2020). Osteocyte-Related Cytokines Regulate Osteoclast Formation and Bone Resorption. Int. J. Mol. Sci..

[28] Vahidi G., Moody M., Welhaven H.D., Davidson L., Rezaee T., Behzad R., Karim L., Roggenbeck B.A., Walk S.T., Martin S.A. (2023). Germ-Free C57BL/6 Mice Have Increased Bone Mass and Altered Matrix Properties but Not Decreased Bone Fracture Resistance. J. Bone Miner. Res..

[29] Li J., Sakisaka Y., Nemoto E., Maruyama K., Suzuki S., Xiong K., Tada H., Tenkumo T., Yamada S. (2024). Cementocyte-derived extracellular vesicles regulate osteoclastogenesis and osteoblastogenesis. J. Dent. Sci..

[30] Sies H. (2015). Oxidative stress: A concept in redox biology and medicine. Redox. Biol..

[31] Singh A., Kukreti R., Saso L., Kukreti S. (2022). Mechanistic Insight into Oxidative Stress-Triggered Signaling Pathways and Type 2 Diabetes. Molecules.

[32] Yaribeygi H., Mohammadi M.T., Sahebkar A. (2018). Crocin potentiates antioxidant defense system and improves oxidative damage in liver tissue in diabetic rats. Biomed. Pharmacother..

[33] Yaribeygi H., Lhaf F., Sathyapalan T., Sahebkar A. (2019). Effects of novel antidiabetes agents on apoptotic processes in diabetes and malignancy: Implications for lowering tissue damage. Life Sci..

[34] Xu N., Liu S., Zhang Y., Chen Y., Zuo Y., Tan X., Liao B., Li P., Feng J. (2023). Oxidative stress signaling in the pathogenesis of diabetic cardiomyopathy and the potential therapeutic role of antioxidant naringenin. Redox. Rep..

[35] Mody N., Parhami F., Sarafian T.A., Demer L.L. (2001). Oxidative stress modulates osteoblastic differentiation of vascular and bone cells. Free Radic. Biol. Med..

[36] Bai X.C., Lu D., Bai J., Zheng H., Ke Z.Y., Li X.M., Luo S.Q. (2004). Oxidative stress inhibits osteoblastic differentiation of bone cells by ERK and NF-κB. Biochem. Biophys. Res. Commun..

[37] Tao Z.S., Wu X.J., Yang M., Shen C.L. (2024). Astaxanthin prevents bone loss in osteoporotic rats with palmitic acid through suppressing oxidative stress. Redox. Rep..

[38] Fatokun A.A., Stone T.W., Smith R.A. (2006). Hydrogen peroxide-induced oxidative stress in MC3T3-E1 cells: The effects of glutamate and protection by purines. Bone.

[39] Chen X., Li Y., Zhang Z., Chen L., Liu Y., Huang S., Zhang X. (2022). Xianling Gubao attenuates high glucose-induced bone metabolism disorder in MG63 osteoblast-like cells. PLoS ONE.

[40] Starup-Linde J., Hygum K., Langdahl B.L. (2018). Skeletal Fragility in Type 2 Diabetes Mellitus. Endocrinol. Metab..

[41] Rendina-Ruedy E., Graef J.L., Lightfoot S.A., Ritchey J.W., Clarke S.L., Lucas E.A., Smith B.J. (2016). Impaired glucose tolerance attenuates bone accrual by promoting the maturation of osteoblasts: Role of Beclin1-mediated autophagy. Bone Rep..

[42] Kang J., Boonanantanasarn K., Baek K., Woo K.M., Ryoo H.M., Baek J.H., Kim G.S. (2015). Hyperglycemia increases the expression levels of sclerostin in a reactive oxygen species- and tumor necrosis factor-alpha-dependent manner. J. Periodontal. Implant. Sci..

[43] Amber R.S., Matthew M.S., Mark E.V.D., Katharina J., Matthew P., Lynda F.B. (2012). Isolation and culture of primary osteocytes from the long bones of skeletally mature and aged mice. Biotechniques.

[44] Yann S., Olivia L. (2023). Endothelial Autophagy Dysregulation in Diabetes. Cells.

[45] Jiang S., Li H., Zhang L., Mu W., Zhang Y., Chen T., Wu J., Tang H., Zheng S., Liu Y. (2024). Generic Diagramming Platform (GDP): A comprehensive database of high-quality biomedical graphics. Nucleic Acids Res..

[46] Forbes J.M., Cooper M.E. (2013). Mechanisms of diabetic complications. Physiol. Rev..

[47] Wu B., Fu Z., Wang X., Zhou P., Yang Q., Jiang Y., Zhu D. (2022). A narrative review of diabetic bone disease: Characteristics, pathogenesis, and treatment. Front. Endocrinol..

[48] Singh H., Singh R., Singh A., Singh H., Singh G., Kaur S., Singh B. (2024). Role of oxidative stress in diabetes-induced complications and their management with antioxidants. Arch. Physiol. Biochem..

[49] Dilworth L., Stennett D., Facey A., Omoruyi F., Mohansingh S., Omoruyi F.O. (2024). Diabetes and the associated complications: The role of antioxidants in diabetes therapy and care. Biomed. Pharmacother..

[50] Lee H.J., Kim D., Do K., Yang C.B., Jeon S.W., Jang A. (2024). Effects of Horse Meat Hydrolysate on Oxidative Stress, Proinflammatory Cytokines, and the Ubiquitin-Proteasomal System of C2C12 Cells. Food Sci. Anim. Resour..

[51] Sies H., Jones D.P. (2020). Reactive oxygen species (ROS) as pleiotropic physiological signalling agents. Nat. Rev. Mol. Cell. Biol..

[52] Schroder K. (2020). NADPH oxidases: Current aspects and tools. Redox. Biol..

[53] Wada J., Makino H. (2013). Inflammation and the pathogenesis of diabetic nephropathy. Clin. Sci..

[54] Ling X., Wang C., Feng Q., Zhang T. (2024). Interleukin-17 prevents oxidative stress from damaging osteoblast formation by inhibiting autophagic degradation of metallothionein-2. Endocr. J..

[55] Feng Y., Yang D., Zhi X., Deng H., Zhang W., Wang R., Wu W. (2022). Role of interaction between reactive oxygen species and ferroptosis pathway in methylglyoxal-induced injury in mouse embryonic osteoblasts. Nan Fang Yi Ke Da Xue Xue Bao.

[56] Li W., Jiang W.S., Su Y.R., Tu K.W., Zou L., Liao C.R., Wu Q., Wang Z.H., Zhong Z.M., Chen J.T. (2023). PINK1/Parkin-mediated mitophagy inhibits osteoblast apoptosis induced by advanced oxidation protein products. Cell Death Dis..

[57] Gao J., Wang Z., Gao P., Fan Q., Zhang T., Cui L., Shi L., Liu Z., Yang Z., He L. (2022). Daphnetin Alleviates Senile and Disuse Osteoporosis by Distinct Modulations of Bone Formation and Resorption. Antioxidants.

[58] Cao F., Yang K., Qiu S., Li J., Jiang W., Tao L., Zhu Y. (2023). Metformin reverses oxidative stress-induced mitochondrial dysfunction in pre-osteoblasts via the EGFR/GSK-3beta/calcium pathway. Int. J. Mol. Med..

[59] Ma X.Y., Cui D., Wang Z., Liu B., Yu H.L., Yuan H., Xiang L.B., Zhou D.P. (2022). Silk Fibroin/Hydroxyapatite Coating Improved Osseointegration of Porous Titanium Implants under Diabetic Conditions via Activation of the PI3K/Akt Signaling Pathway. ACS Biomater. Sci. Eng..

[60] Li Y., Zhang J., Li F. (2024). Gastrodin improves osteoblast function and adhesion to titanium surface in a high glucose environment. Biochem. Biophys. Rep..

[61] Mahboob A., Senevirathne D.K.L., Paul P., Nabi F., Khan R.H., Chaari A. (2023). An investigation into the potential action of polyphenols against human Islet Amyloid Polypeptide aggregation in type 2 diabetes. Int. J. Biol. Macromol..

[62] Rehan Ahmad S., Zeyaullah M., AlShahrani A.M., Qavi D., Altijani A.A.G., Khan M.S., Muzammil K. (2025). An Unstable ATG2A Variant Causes a Neurodegenerative Disorder via Impaired Autophagy and Proteotoxic Stress in Brain Atrophy. Clin. Genet..

[63] Alexander A., Cai S.L., Kim J., Nanez A., Sahin M., MacLean K.H., Inoki K., Guan K.L., Shen J., Person M.D. (2010). ATM signals to TSC2 in the cytoplasm to regulate mTORC1 in response to, R.O.S. Proc. Natl. Acad. Sci. USA.

[64] Kim Y.C., Guan K.L. (2015). mTOR: A pharmacologic target for autophagy regulation. J. Clin. Invest..

[65] Bou-Teen D., Kaludercic N., Weissman D., Turan B., Maack C., Di Lisa F., Ruiz-Meana M. (2021). Mitochondrial ROS and mitochondria-targeted antioxidants in the aged heart. Free Radic. Biol. Med..

[66] Kalkavan H., Green D.R. (2018). MOMP, cell suicide as a BCL-2 family business. Cell Death Differ..

[67] Jiang X., Wang X. (2004). Cytochrome C-mediated apoptosis. Annu. Rev. Biochem..

[68] Redza-Dutordoir M., Averill-Bates D.A. (2016). Activation of apoptosis signalling pathways by reactive oxygen species. Biochim. Biophys. Acta.

[69] Han R.H., Huang H.M., Han H., Chen H., Zeng F., Xie X., Liu D.Y., Cai Y., Zhang L.Q., Liu X. (2021). Propofol postconditioning ameliorates hypoxia/reoxygenation induced H9c2 cell apoptosis and autophagy via upregulating forkhead transcription factors under hyperglycemia. Mil. Med. Res..

[70] Zhao Z., Ming Y., Li X., Tan H., He X., Yang L., Song J., Zheng L. (2023). Hyperglycemia Aggravates Periodontitis via Autophagy Impairment and ROS-Inflammasome-Mediated Macrophage Pyroptosis. Int. J. Mol. Sci..

[71] Demirtas L., Guclu A., Erdur F.M., Akbas E.M., Ozcicek A., Onk D., Turkmen K. (2016). Apoptosis, autophagy & endoplasmic reticulum stress in diabetes mellitus. Indian J. Med. Res..

[72] Gao G., Chen W., Yan M., Liu J., Luo H., Wang C., Yang P. (2020). Rapamycin regulates the balance between cardiomyocyte apoptosis and autophagy in chronic heart failure by inhibiting mTOR signaling. Int. J. Mol. Med..

[73] Eastell R., Szulc P. (2017). Use of bone turnover markers in postmenopausal osteoporosis. Lancet Diabetes Endocrinol..

[74] Rahmani D., Faal B., Zali H., Tackallou S.H., Niknam Z. (2023). The beneficial effects of simultaneous supplementation of Lactobacillus reuteri and calcium fluoride nanoparticles on ovariectomy-induced osteoporosis. BMC Complement. Med. Ther..

[75] Zhang X., Huang F., Chen X., Wu X., Zhu J. (2020). Ginsenoside Rg3 attenuates ovariectomy-induced osteoporosis via AMPK/mTOR signaling pathway. Drug. Dev. Res..

[76] Li Y.R., Li S., Lin C.C. (2018). Effect of resveratrol and pterostilbene on aging and longevity. Biofactors.

