# Supplementary material for: Reactive Oxygen Species (ROS) Drive Osteocyte Dysfunction in Diabetic Osteoporosis by Impairing Autophagy and Triggering Apoptosis
Source: Antioxidants (Basel). 2025 Oct 30;14(11):1306. doi: 10.3390/antiox14111306 (PMC12649596; doi:10.3390/antiox14111306)
Supplement: Supplementary file 1 [file antioxidants-14-01306-s001.zip › antioxidants-3823597-supplementary.pdf]

a

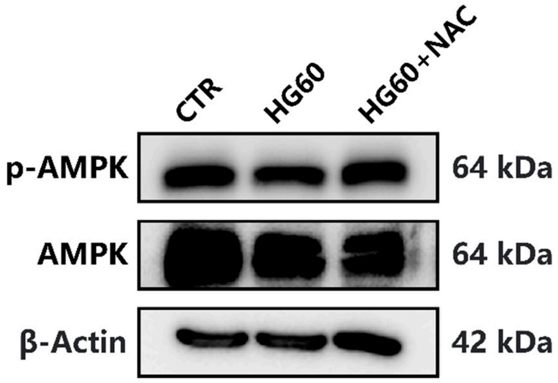

b

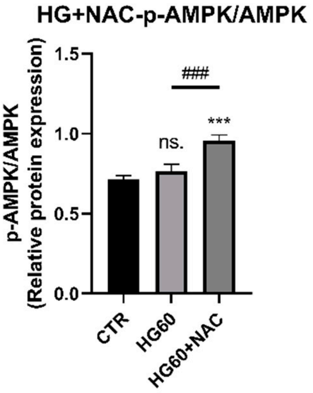

Supplementary Figure S1. Representative Western blot bands and protein quantification of p-AMPK and AMPK in MLO-Y4 cells. CTR: Normal glucose. HG60: 60 mmol/L glucose. HG60+NAC: 60 mmol/L glucose with 8 mmol/L NAC. β-Actin was used as the loading control. Data are presented as mean ± SEM (biological replicates, n = 3). *** $p < 0.001$ vs. CTR, ### $p < 0.001$ vs. HG60.
